# Association of handgrip strength and risk of cardiovascular disease: a population-based cohort study

**DOI:** 10.1007/s40520-024-02856-x

**Published:** 2024-10-16

**Authors:** Fan Zhang, Bingru Luo, Yan Bai, Ying Zhang, Liuyan Huang, Wei Lu

**Affiliations:** 1 Department of Nephrology A, Longhua Hospital Shanghai University of Traditional Chinese Medicine, Shanghai, China; 2 Department of Neurological Critical Care Medicine, West China Hospital, Sichuan University, Sichuan, China; 3 Department of Surgery, Longhua Hospital Shanghai University of Traditional Chinese Medicine, Shanghai, China; 4 Department of Nursing, Longhua Hospital Shanghai University of Traditional Chinese Medicine, No. 725, Wanping South Road, Xuhui District, Shanghai, China

**Keywords:** Handgrip strength, CHARLS, Cardiovascular disease, Heart disease, Stroke

## Abstract

**Background:**

Handgrip strength (HGS) is a simple and non-invasive measure of muscle strength, which has been proposed as a potential predictor of cardiovascular disease (CVD) risk. However, the association between HGS and CVD risk in the Chinese population remains underexplored. This study aims to investigate the relationship between handgrip strength and the risk of cardiovascular disease using data from the 2015–2018 China Health and Retirement Longitudinal Study (CHARLS).

**Methods:**

We included 9369 participants aged 45 years and older from the CHARLS dataset. HGS was measured using a dynamometer, and the average of three measurements for each hand was recorded. Participants were categorized into quartile based on their HGS levels. The primary outcome was the incidence of CVD, including heart diseases, and stroke, obtained through self-reports and verified by medical records. Cox proportional hazards models were used to estimate hazard ratio (HR) and 95% confidence interval (95% CI) for the association between HGS and CVD risk, and using restricted cubic spline to construct a linear relationship, adjusting for potential covariates including demographic factors, lifestyle factors, body composition, and health status. The robustness of the results was determined by stratified analysis.

**Results:**

During a mean follow-up of 3 years, 1198 CVD events were reported, including 851 heart diseases and 427 strokes. After multivariate adjustment, the HR and 95% CI corresponding to CVD risk for participants in the HGS 2nd, 3rd, and 4th quartiles compared with those in the lowest quartile were 0.824 (95% CI: 0.692–0.981), 0.756 (95% CI: 0.614–0.929), 0.625 (95% CI: 0.484–0.807) (*P*_for trend_: <0.001), respectively. All subgroups analyzed found similar results. For CVD components, HGS was similarly linearly associated with heart disease, and had an inverted U-shape relationship with the risk of stroke, with a potential threshold of 23.16 kg.

**Conclusions:**

Higher HGS was significantly associated with decreased risk of CVD, including heart disease and stroke, in middle-aged and older adults, suggesting that HGS may be a promising measurable predictor of CVD in this population.

**Supplementary Information:**

The online version contains supplementary material available at 10.1007/s40520-024-02856-x.

## Introduction

Cardiovascular disease (CVD) is the leading cause of death globally, and it is estimated that about 17.9 million people died from CVD globally in 2019, accounting for 32% of all deaths [1]. CVD not only causes tremendous loss of life but also places a heavy burden on healthcare systems and socioeconomics [2, 3]. Therefore, prevention of CVD is essential to improve population health and reduce the burden of disease.

Handgrip strength (HGS) is a simple, non-invasive, and cost-effective measure of muscle strength that has gained attention as a potential predictor of various health outcomes, including mortality, disability, and chronic diseases [4–7]. Previous studies have shown that HGS is strongly associated with cardiovascular health. A prospective cohort study that included nearly 140,000 healthy adults from 17 countries found that for every 5 kg decrease in HGS, the risk of all-cause mortality, cardiovascular mortality, and cardiovascular events increased by 16%, 17%, and 7%, respectively [8]. A study by Ortega et al. [9] came to a similar conclusion, that a greater HGS in adolescence was associated with a lower risk of CVD in adulthood. These findings support that HGS may be an ideal predictor of CVD risk.

Although the association between HGS and CVD has been examined in many studies, there are still some issues to be further explored. First, most studies come from Europe and the United States, and there is a paucity of evidence from Asian populations, especially Chinese populations [10]. Considering the differences in ethnicity, body size, and lifestyle, it is necessary to conduct relevant studies on the Chinese population, to provide a reference for the development of CVD prevention and control strategies suitable for the Chinese situation.

Second, most previous studies have focused only on the dichotomous association between HGS and CVD (i.e., lower vs. higher HGS group), while ignoring the possible nonlinear relationship between the two [11]. There are several reasons to suspect a nonlinear relationship between HGS and CVD risk. From a physiological perspective, extremely low HGS may reflect sarcopenia or poor overall health, while extremely high HGS might be associated with certain metabolic abnormalities or overtraining, both of which could potentially increase CVD risk [12]. Furthermore, the relationship between HGS and CVD risk may vary across different age groups and between sexes, potentially leading to a nonlinear relationship in the overall population [13]. Additionally, other health indicators such as body mass index (BMI) and blood pressure have shown nonlinear relationships with disease risks [14], suggesting that HGS might follow a similar pattern.

In addition, it is unclear whether there is a specific effect of HGS on different types of CVD (e.g., heart disease and stroke) [15]. Elucidating these issues will contribute to a more comprehensive understanding of the relationship between HGS and cardiovascular health. Based on the above research background, the present study, using data from the 2015–2018 China Health and Retirement Longitudinal Study (CHARLS), aimed to (1) assess the association between HGS and risk of CVD development; (2) explore the nonlinear relationship for both; and (3) analyze the potential effect modifying role of age, gender, BMI and other factors.

## Methods

This study followed STrengthening the Reporting of OBservational studies in Epidemiology (STROBE) reporting guidelines (Table S1) [16].

### Study population

The data used in this study were obtained from CHARLS, a national longitudinal survey designed to be representative of China’s population aged 45 years and older. CHARLS utilized a multi-stage, stratified, probability sampling design proportional to population size, covering urban and rural areas in 31 provinces in mainland China [17]. The survey randomly selected 450 village committees/habitat committees out of 150 county-level units, ensuring a geographically diverse sample that included both economically developed and less developed areas in the east, center, and west. Our study used data from 2015 as a baseline, with a follow-up to 2018. Although CHARLS aims to provide a nationally representative sample, we recognize that there may be limitations, such as the possibility of not fully representing the institutionalized elderly population or the critically ill.

**Fig. 1:**
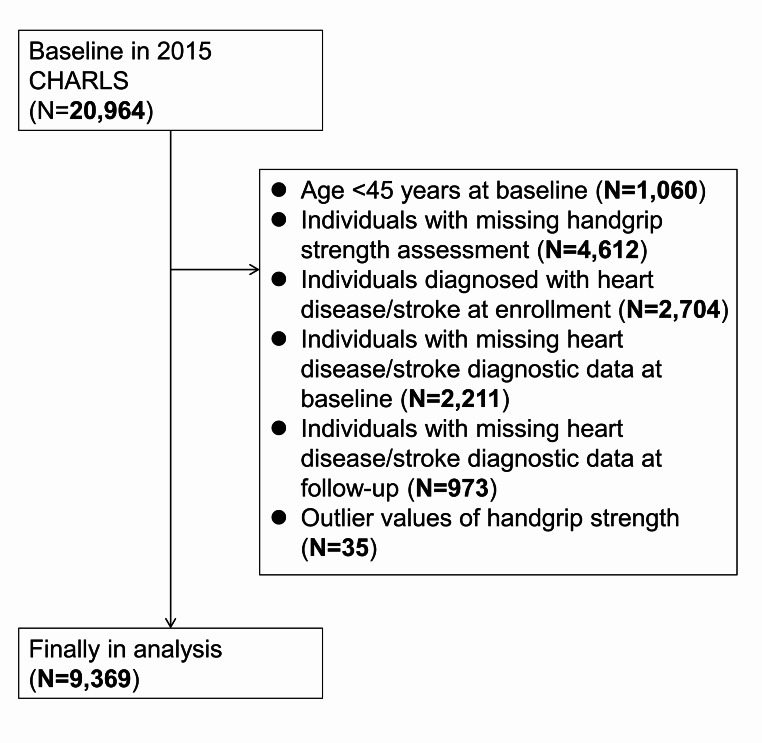
Participants screen flowchart

Subjects who participated in the 2015 survey with complete HGS data were included in this study. Based on the 2015 population (*n* = 20,964), participants with baseline preexisting CVD and those with HGS deficits were excluded, and participants aged < 45, as well as those with missing or outlier values of key variables (HGS < 5 kg or > 70 kg), were excluded, finally, 9,369 middle-aged and older adults were included in the analysis, Fig. 1 depicts the study population screening process. During the 2018 follow-up, information about CVD incidence was obtained through family visits. All participants signed an informed consent form, and the study protocol was approved by the Ethics Committee of Peking University (approval number: IRB00001052-11,015).

### Exposure

HGS was measured in kilograms by trained volunteers using a YuejianTM WL-1000 dynamometer (Nantong Yuejian Physical Measurement Instrument Co., Ltd., Nantong, China) [17]. Subjects stood and started the test using either the dominant or non-dominant hand while receiving verbal encouragement. Each subject held the ergometer at a right angle (90°) and squeezed the handle for a few seconds, taking two measurements on the right and left hand. Participants were asked to provide maximum effort to perform the measurements [18]. The higher value between the two hands was used for analysis. To explore the relationship between HGS and CVD risk, we categorized HGS into quartiles according to the distribution of our study population. This categorization allowed us to explore the impact of HGS levels relative to our study population without relying on external criteria.

### Outcomes

The primary outcome was CVD events, including heart disease and stroke. Similar to previous studies [19], CVD events were assessed by the following questions: “Have you been told by a doctor that you have been diagnosed with a heart attack, angina, coronary heart disease, heart failure, or other heart problems?” or “Have you been told by a doctor that you have been diagnosed with a stroke?“. Participants who reported having a heart disease or stroke were defined as having CVD.

### Covariates

Potential confounders were selected based on previous literature and their relevance to both HGS and CVD risk. These included:

Demographic factors: age (continuous; <60, >=60), gender (male, female), education level (elementary school or below, secondary school, college and above), marriage (marriage, other), and residence (urban, rural).

Body composition: BMI (underweight, normal, overweight or obesity), muscle mass (low, normal).

Lifestyle factors: smoking status (current, former, never), alcohol consumption (current, former, never).

Health status: physical performance (ADL/IADL without loss, ADL/IADL loss), history of hypertension (yes, no), diabetics (yes, no), kidney disease (yes, no), and dyslipidemia (yes, no).

Where muscle mass is defined as the ratio of appendicular skeletal muscle mass (ASM) to height squared (i.e. ASM/Ht^2^). According to Gao et al. [20], the threshold value for the definition of low muscle mass was based on the ASM/Ht^2^ of the lowest 20% percentile of study population. Therefore, females with an ASM/Ht^2^ value of < 5.59 kg/m^2^ and males with an ASM/Ht^2^ value of < 7.22 kg/m^2^ were defined as having low muscle mass. Physical performance was assessed using Activities of Daily Living (ADL) and Instrumental Activity of Daily Living (IADL), which included bathing, eating, dressing, getting in and out of bed, toileting and defecation, cooking, housework, medication management, telephone communication, shopping, and financial management. There are questions for each of these items, and IADL typically uses six items for its assessments. Each question contained four responses: “Have any difficulty,” “have difficulty but can still do it,” “have difficulty and need help,” and “cannot do it.” If the subject completed all of the items without “having any difficulty,” we defined this as “ADL/IADL without loss.” Conversely, if the subject completed any of the items “with any difficulty,” we categorized it as “ADL/IADL Loss.”

### Statistical analysis

Descriptive statistics were used to summarize the baseline characteristics of the study population across quartile of HGS. Differences between groups were assessed using chi-square tests for categorical variables and ANOVA for continuous variables. Cox proportional hazard models were used to estimate hazard ratio (HR) and 95% confidence interval (95% CI) for the association between HGS and incident CVD. Follow-up time was calculated from the date of the baseline survey (2015) to the date of CVD diagnosis or the last follow-up visit, whichever occurred first. For participants who died during the follow-up period without experiencing a CVD event, the date of death was considered as the end of follow-up. The proportional hazards assumption was tested using Schoenfeld residuals. The models were adjusted for potential confounders in a stepwise manner:

1. Unadjusted model.
2. Model adjusted for demographic factors (age, sex, education level, marriage, and residence).
3. Model further adjusted for lifestyle factors (smoking status, alcohol consumption), body composition (muscle mass, BMI), and physical performance.
4. Fully adjusted model including demographic factors, body composition, lifestyle factors, and health status.

Next, a multivariate restricted cubic spline model was used to evaluate a potential non-linear relationship between the HGS and CVD. We selected three components at the 25th, 50th, and 75th quartiles. The nonlinearity was tested using a likelihood ratio test, comparing the model with only the linear term to the model with both the linear and the cubic spline terms. A *P*-value < 0.05 from this likelihood ratio test was considered as evidence of a nonlinear relationship. Subgroup analyses were conducted to examine the consistency of the association across different characteristics. All statistical analyses were performed using R (version 4.2.0). A two-sided *P*-value of < 0.05 was considered statistically significant.

## Result

A total of 9369 participants were included in the analysis. The mean age of participants was 56.69 years (standard deviation 9.0 years). There are 4661 participants (49.7%) were male. The median HGS was 30.50 kg (interquartile range: 24.15–38.50 kg). Table 1 summarizes the baseline characteristics based on the HGS quartiles.

**Table 1:** The baseline characteristics based on HGS quartiles

Characteristic	Overall	Q1 (< 24.15 kg)	Q2 (24.15–30.49 kg)	Q3 (30.50–38.49 kg)	Q4 ( > = 38.50 kg)	*P*-value
Participants	*N* = 9,369	*N* = 2,339	*N* = 2,314	*N* = 2,369	*N* = 2,347	
HGS, Median (IQR)	30.50 (24.15, 38.50)	20.25 (17.45, 22.50)	27.30 (25.75, 28.80)	34.00 (32.00, 36.00)	43.20 (40.50, 47.00)	< 0.001
Age, years	56.00 (49.00, 62.00)	60.00 (53.00, 68.00)	56.00 (49.00, 62.00)	55.00 (48.00, 62.00)	51.00 (47.00, 58.00)	< 0.001
Age category, n (%)						< 0.001
Adults (< 60)	6100 (65.1%)	1100 (47.0%)	1514 (65.4%)	1586 (66.9%)	1900 (81.0%)	
Elderly ( > = 60)	3269 (34.9%)	1239 (53.0%)	800 (34.6%)	783 (33.1%)	447 (19.0%)	
Gender, n (%)						< 0.001
Male	4,661.0 (49.7%)	282.0 (12.1%)	601.0 (26.0%)	1,526.0 (64.4%)	2,252.0 (96.0%)	
Female	4,708.0 (50.3%)	2,057.0 (87.9%)	1,713.0 (74.0%)	843.0 (35.6%)	95.0 (4.0%)	
Education, n (%)						< 0.001
Elementary school or below	2,068.0 (24.3%)	963.0 (43.5%)	602.0 (28.2%)	357.0 (16.6%)	146.0 (7.2%)	
Secondary school	5,844.0 (68.7%)	1,178.0 (53.3%)	1,412.0 (66.1%)	1,617.0 (75.4%)	1,637.0 (81.2%)	
College and above	597 (7.0%)	71 (3.2%)	123 (5.8%)	171 (8.0%)	232 (11.5%)	
ASM/height^2^, Median (IQR)	7.06 (6.13, 7.76)	6.01 (5.49, 6.61)	6.50 (5.98, 7.17)	7.34 (6.69, 7.85)	7.92 (7.48, 8.38)	< 0.001
Muscle mass, n (%) ^b^						< 0.001
Low muscle mass	1875 (20.0%)	866 (37.0%)	415 (17.9%)	367 (15.5%)	227 (9.7%)	
Normal	7494 (80.0%)	1473 (63.0%)	1899 (82.1%)	2002 (84.5%)	2120 (90.3)	
Physical perform, n (%)						< 0.001
ADL/IADL without loss	7,271.0 (77.6%)	1,674.0 (71.6%)	1,799.0 (77.7%)	1,825.0 (77.0%)	1,973.0 (84.1%)	
ADL/IADL loss	2,098.0 (22.4%)	665.0 (28.4%)	515.0 (22.3%)	544.0 (23.0%)	374.0 (15.9%)	
Marriage, n (%)						< 0.001
Married	8,341.0 (89.0%)	1,870.0 (79.9%)	2,055.0 (88.8%)	2,167.0 (91.5%)	2,249.0 (95.8%)	
Other	1,028.0 (11.0%)	469.0 (20.1%)	259.0 (11.2%)	202.0 (8.5%)	98.0 (4.2%)	
BMI, km/m^2^	23.89 (21.77, 26.28)	23.32 (21.07, 25.77)	24.03 (22.10, 26.52)	23.74 (21.74, 26.08)	24.38 (22.13, 26.65)	< 0.001
BMI category, n (%)						< 0.001
Underweight ( < = 18.4)	304 (3.2%)	186 (8.0%)	76 (3.3%)	34 (1.4%)	8 (0.3%)	
Normal (18.5–23.9)	4393 (46.9%)	1121 (47.9%)	1039 (44.9%)	1194 (50.4%)	1039 (44.3%)	
Overweight or obesity ( > = 24.0)	4672 (49.9%)	1032 (44.1%)	1199 (51.8%)	1141 (48.2%)	1300 (55.4%)	
Drinking, n (%)						< 0.001
Never smoked	2,674.0 (28.6%)	285.0 (12.2%)	390.0 (16.9%)	778.0 (32.9%)	1,221.0 (52.1%)	
Currently smoking	5,891.0 (63.0%)	1,887.0 (80.9%)	1,767.0 (76.6%)	1,341.0 (56.7%)	896.0 (38.2%)	
Quit drinking	784.0 (8.4%)	161.0 (6.9%)	150.0 (6.5%)	247.0 (10.4%)	226.0 (9.6%)	
Smoking, n (%)						< 0.001
Never smoked	5,480.0 (58.5%)	1,956.0 (83.7%)	1,784.0 (77.1%)	1,161.0 (49.0%)	579.0 (24.7%)	
Currently smoking	2,755.0 (29.4%)	270.0 (11.6%)	362.0 (15.6%)	839.0 (35.4%)	1,284.0 (54.8%)	
Quit smoking	1,129.0 (12.1%)	111.0 (4.7%)	168.0 (7.3%)	368.0 (15.5%)	482.0 (20.6%)	
Residence, n (%)						< 0.001
Urban	1,865.0 (19.9%)	371.0 (15.9%)	452.0 (19.5%)	470.0 (19.8%)	572.0 (24.4%)	
Rural	7,504.0 (80.1%)	1,968.0 (84.1%)	1,862.0 (80.5%)	1,899.0 (80.2%)	1,775.0 (75.6%)	
Hypertension, n (%)						0.01
No	5,589.0 (59.8%)	1,325.0 (56.8%)	1,404.0 (60.8%)	1,430.0 (60.4%)	1,430.0 (61.0%)	
Yes	3,762.0 (40.2%)	1,007.0 (43.2%)	905.0 (39.2%)	936.0 (39.6%)	914.0 (39.0%)	
Systolic blood pressure, mmHg	125.67 (114.00, 140.00)	126.33 (113.33, 141.00)	124.33 (112.67, 139.00)	126.33 (114.33, 140.33)	126.00 (115.67, 138.67)	0.003
Diastolic blood pressure, mmHg	75.00 (68.00, 82.67)	73.00 (66.33, 80.67)	73.83 (67.00, 81.67)	75.67 (68.67, 83.33)	77.67 (70.33, 85.67)	< 0.001
Diabetics, n (%)						0.287
No	7,296.0 (93.4%)	1,803.0 (92.9%)	1,837.0 (92.8%)	1,854.0 (93.9%)	1,802.0 (94.1%)	
Yes	514.0 (6.6%)	137.0 (7.1%)	142.0 (7.2%)	121.0 (6.1%)	114.0 (5.9%)	
Dyslipidemia, n (%)						< 0.001
No	5,391.0 (69.0%)	1,368.0 (70.8%)	1,424.0 (71.7%)	1,371.0 (69.3%)	1,228.0 (64.0%)	
Yes	2,425.0 (31.0%)	564.0 (29.2%)	563.0 (28.3%)	608.0 (30.7%)	690.0 (36.0%)	
Kidney disease, n (%)						< 0.001
No	6,323.0 (80.9%)	1,468.0 (76.1%)	1,644.0 (82.8%)	1,622.0 (82.0%)	1,589.0 (82.6%)	
Yes	1,492.0 (19.1%)	460.0 (23.9%)	342.0 (17.2%)	356.0 (18.0%)	334.0 (17.4%)	

^a^ Missing data: 860 for education, 20 for drinking, 5 for smoking, 18 for hypertension, 36 for systolic blood pressure, 38 for diastolic blood pressure, 1559 for diabetics, 1553 for dyslipidemia, 1554 for kidney disease

^b^ Muscle mass, assessed by ASM/Ht^2^

^c^ Q, quartile; HGS, handgrip strength; IQR, interquartile range; ASM/Ht^2^, height-adjusted muscle mass; BMI, body mass index

During the 28,107 person-years of follow-up, there were 1,198 CVD events recorded, including 851 heart disease cases and 427 strokes. The incidence of CVD located in HGS 1st quartile participants was 53.727 cases per 1000 person-years, 2nd quartile participants 43.215 cases, 3rd quartile participants 39.820 cases, and 4th quartile participants 33.802 cases (Table 2). After adjustment for covariates, 2nd quartile, 3rd quartile, and 4th participants had a 17.6 (HR: 0.824; 95% CI: 0.692–0.981), 24.4% (HR: 0.756; 95% CI: 0.614–0.929), and 37.5% (HR: 0.625; 95% CI: 0.484–0.807) reduction in risk for CVD incidence during the follow-up period, respectively, as compared to participants with lowest quartile (*P*_for trend_: <0.001). For every 5 kg increase in HGS, the risk of CVD was reduced by 7.2% (HR: 0.928; 95% CI: 0.883–0.975) (Table 2).

**Table 2:** Incidence of CVD according to baseline HGS status, 2015 − 2018

Outcomes			Model 1	Model 2	Model 3	Model 4
CVD	Case	Incidence Rate, per 1000 Person-Years	HR (95% CI)	HR (95% CI)	HR (95% CI)	HR (95% CI)
Per 5 kg increase			0.920 (0.893–0.947)	0.964 (0.924–1.007)	0.928 (0.887–0.972)	0.928 (0.883–0.975)
HGS quartile						
Q1 (< 24.15 kg)	377	53.727	Ref.	Ref.	Ref.	Ref.
Q2 (24.15–30.49 kg)	300	43.215	0.790 (0.679–0.919)	0.866 (0.737–1.017)	0.810 (0.688–0.954)	0.824 (0.692–0.981)
Q3 (30.50–38.49 kg)	283	39.820	0.724 (0.621–0.845)	0.813 (0.674–0.981)	0.723 (0.596–0.877)	0.756 (0.614–0.929)
Q4 ( > = 38.50 kg)	238	33.802	0.608 (0.517–0.715)	0.739 (0.587–0.929)	0.631 (0.498-0.800)	0.625 (0.484–0.807)
*P* for trend			< 0.001	0.008	< 0.001	< 0.001
Heart disease						
Per 5 kg increase			0.908 (0.876–0.940)	0.862 (0.913–1.014)	0.926 (0.877–0.979)	0.919 (0.865–0.976)
HGS quartile						
Q1 (< 24.15 kg)	279	40.069	Ref.	Ref.	Ref.	Ref.
Q2 (24.15–30.49 kg)	216	31.250	0.765 (0.641–0.914)	0.824 (0.682–0.996)	0.784 (0.647–0.949)	0.775 (0.631–0.952)
Q3 (30.50–38.49 kg)	197	27.801	0.682 (0.568–0.819)	0.802 (0.643–1.002)	0.725 (0.577–0.911)	0.754 (0.591–0.963)
Q4 ( > = 38.50 kg)	159	22.640	0.551 (0.453–0.669)	0.704 (0.533–0.931)	0.600 (0.450–0.800)	0.567 (0.414–0.775)
*P* for trend			< 0.001	0.012	< 0.001	< 0.001
Stroke						
Per 5 kg increase			0.944 (0.899–0.991)	0.950 (0.887–1.018)	0.915 (0.850–0.984)	0.922 (0.852–0.998)
HGS quartile						
Q1 (< 24.15 kg)	125	17.829	Ref.	Ref.	Ref.	Ref.
Q2 (24.15–30.49 kg)	104	14.988	0.837 (0.645–1.085)	0.913 (0.694–1.201)	0.831 (0.629–1.097)	0.877 (0.654–1.175)
Q3 (30.50–38.49 kg)	110	15.491	0.867 (0.671–1.120)	0.826 (0.606–1.126)	0.706 (0.512–0.974)	0.706 (0.501–0.995)
Q4 ( > = 38.50 kg)	88	12.504	0.696 (0.530–0.914)	0.690 (0.478–0.995)	0.585 (0.400-0.856)	0.616 (0.412–0.922)
*P* for trend			0.017	0.048	0.005	0.014

Model 1: Unadjusted

Model 2: Adjusted for age, gender, education, marriage, and residence

Model 3: Adjusted for Model 2 + muscle mass, physical perform, BMI, drinking, smoking

Model 4: Adjusted for Model 3 + history of hypertension, dyslipidemia, diabetes, kidney disease

HR, hazard ratio; Q, quartile; CVD, cardiovascular disease; 95% CI, 95% confidence interval

For CVD components, in fully adjusted models including sociodemographic information, lifestyles, body composition, and health status, the multivariate-adjusted HRs and 95% CIs for participants located in the HGS 2nd, 3rd, 4th quartile participant were: heart disease: 0.775 (95% CI: 0.631–0.952); 0.754 (95% CI: 0.591–0.963); 0.567 (95% CI: 0.414–0.775) (*P*_for trend_: <0.001), stroke: 0.877 (95% CI: 0.654–1.175); 0.706 (95% CI: 0.501–0.995); 0.616 (95% CI: 0.412–0.922) (*P*_for trend_: 0.014). For every 5 kg increase in HGS, the risk of heart disease and stroke was reduced by 8.1% (HR: 0.919; 95% CI: 0.865–0.976), and 7.3% (HR:0.922; 95% CI: 0.852–0.998), respectively.

**Fig. 2:**
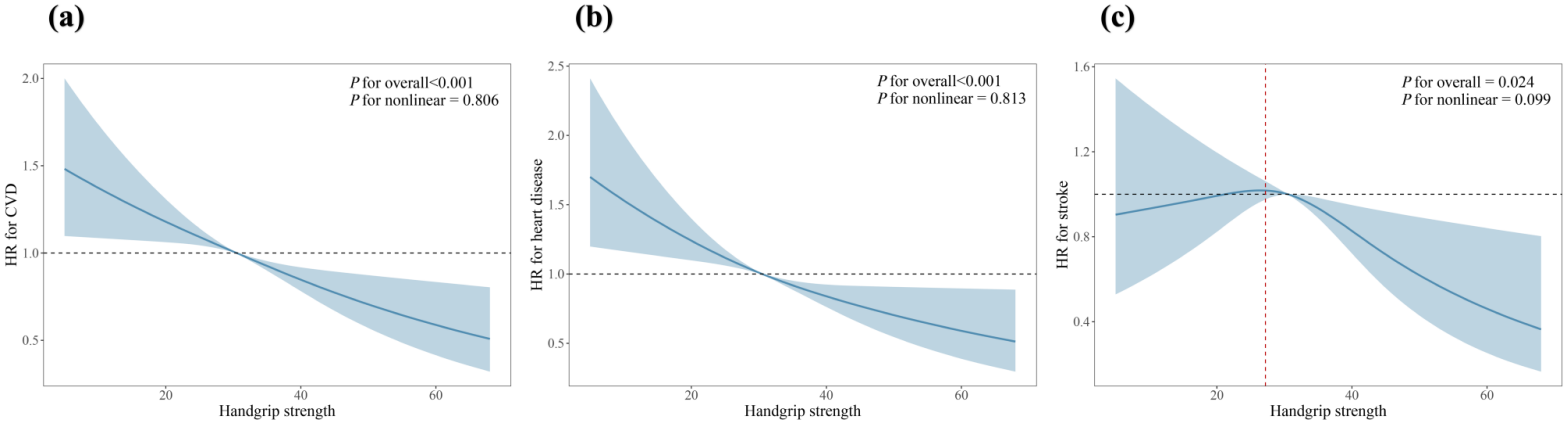
Association between HGS with CVD among middle-aged and elderly people in CHARLS 2015–2018. HRs were adjusted for age (< 60 or > = 60), gender (male or female), education (“elementary school or below” or secondary school or “college and above”), marriage (married or other), residence (urban or rural), muscle mass (low muscle mass or normal), physical perform (“ADL/IADL without loss” or “ADL/IADL loss”), BMI (underweight or normal or “overweight or obesity”), drinking (never smoked or currently smoking or quit drinking), smoking (never smoked or currently smoking or quit drinking), history of hypertension (no or yes), dyslipidemia (no or yes), diabetes (no or yes), and kidney disease (no or yes)

As shown in Fig. 2a **and** Fig. 2b, a linear relationship between HGS and the risk of CVD and heart disease was found by restricted cubic spline analysis combined with Cox proportional hazards modeling, i.e., as HGS increased, the risk of CVD and heart disease decreased. In addition, we found an inverted U-shaped relationship between HGS and stroke risk (Fig. 2c). We found that the inflection point for stroke risk was 26.13 kg. When HGS was above the inflection point, the risk of stroke incidence decreased by 8.3% (HR: 0.917, 95% CI: 0.866–0.971) for every 1 kg increase in HGS, as shown in Table S2.

Stratified analyses showed that no interactions were found for variables other than smoking. In the subgroup aged < 60 years, the HR corresponding to the risk of CVD occurrence in HGS 2nd quartile, 3rd quartile, and 4th quartile participants compared with the reference group was 0.719 (95% CI: 0.562–0.918), 0.701 (95% CI: 0.531–0.926), and 0.551 (95% CI: 0.390–0.777), respectively (*P* for trend: 0.001). Among male participants, the HR corresponding to the risk of CVD occurrence was 0.871 (95% CI: 0.582-1.300), 0.722 (95% CI: 0.492–1.060), and 0.647 (95% CI: 0.433–0.967) for HGS 2nd quartile, 3rd quartile, and 4th quartile participants, respectively, compared with the reference group (*P for trend*: 0.014). As shown in Table 3, the results of the remaining subgroup analyses similarly show effects in the same direction for all groups.

**Table 3:** Stratified analyses of the associations between HGS and CVD among middle-aged and elderly people in CHARLS 2015–2018

Subgroup	Q1	Q2 (HR, 95% CI)	Q3 (HR, 95% CI)	Q4 (HR, 95% CI)	*P*_for trend_	*P*_for interaction_
Age category						0.278
Adults (< 60)	Ref.	0.719 (95% CI 0.562–0.918)	0.701 (95% CI 0.531–0.926)	0.551 (95% CI 0.390–0.777)	0.001	
Elderly ( > = 60)	Ref.	0.933 (95% CI 0.726-1.200)	0.796 (95% CI 0.584–1.090)	0.721 (95% CI 0.489–1.060)	0.076	
Gender						0.289
Male	Ref.	0.871 (95% CI 0.582-1.300)	0.722 (95% CI 0.492–1.060)	0.647 (95% CI 0.433–0.967)	0.014	
Female	Ref.	0.795 (95% CI 0.652–0.968)	0.810 (95% CI 0.623–1.050)	0.745 (95% CI 0.380–1.460)	0.039	
BMI						0.907
Underweight	Ref.	1.210 (95% CI 0.421-3.500)	1.240 (95% CI 0.315–4.870)	0.797 (95% CI 0.072–8.770)	0.897	
Normal	Ref.	0.823 (95% CI 0.619–1.090)	0.683 (95% CI 0.491–0.950)	0.618 (95% CI 0.414–0.923)	0.012	
Overweight or obesity	Ref.	0.808 (95% CI 0.643–1.020)	0.783 (95% CI 0.597–1.030)	0.635 (95% CI 0.451–0.894)	0.011	
Drinking						0.723
Never smoked	Ref.	0.944 (95% CI 0.560–1.590)	0.912 (95% CI 0.524–1.590)	0.725 (95% CI 0.403-1.300)	0.017	
Currently smoking	Ref.	0.817 (95% CI 0.671–0.995)	0.732 (95% CI 0.572–0.935)	0.589 (95% CI 0.413–0.839)	0.001	
Quit drinking	Ref.	0.831 (95% CI 0.455–1.520)	0.760 (95% CI 0.400–1.440)	0.813 (95% CI 0.400–1.650)	0.600	
Smoking						0.032
Never smoked	Ref.	0.873 (95% CI 0.716–1.060)	0.772 (95% CI 0.596–0.999)	0.583 (95% CI 0.379–0.897)	0.009	
Currently smoking	Ref.	0.671 (95% CI 0.413–1.090)	0.729 (95% CI 0.456–1.160)	0.603 (95% CI 0.366–0.992)	0.091	
Quit smoking	Ref.	0.649 (95% CI 0.354–1.190)	0.583 (95% CI 0.316–1.070)	0.517 (95% CI 0.270–0.991)	0.075	
Hypertension						0.456
No	Ref.	0.630 (95% CI 0.475–0.835)	0.705 (95% CI 0.512–0.969)	0.546 (95% CI 0.365–0.815)	0.004	
Yes	Ref.	0.991 (95% CI 0.791–1.240)	0.793 (95% CI 0.605–1.040)	0.697 (95% CI 0.500-0.971)	0.024	
Diabetics						0.704
No	Ref.	0.801 (95% CI 0.665–0.965)	0.746 (95% CI 0.599–0.927)	0.611 (95% CI 0.467-0.800)	< 0.001	
Yes	Ref.	0.980 (95% CI 0.585–1.640)	0.834 (95% CI 0.429–1.620)	0.734 (95% CI 0.316-1.700)	0.459	
Dyslipidemia						0.636
No	Ref.	0.878 (95% CI 0.708–1.090)	0.818 (95% CI 0.632–1.060)	0.736 (95% CI 0.535–1.010)	0.056	
Yes	Ref.	0.726 (95% CI 0.537–0.981)	0.647 (95% CI 0.456–0.919)	0.458 (95% CI 0.297–0.706)	0.001	
Kidney disease						0.657
No	Ref.	0.786 (95% CI 0.644–0.960)	0.719 (95% CI 0.569–0.910)	0.577 (95% CI 0.430–0.774)	< 0.001	
Yes	Ref.	0.956 (95% CI 0.662–1.380)	0.874 (95% CI 0.562–1.360)	0.758 (95% CI 0.445–1.290)	0.320	

Models were adjusted for age (< 60 or > = 60), gender (male or female), education (“elementary school or below” or secondary school or “college and above”), marriage (married or other), residence (urban or rural), muscle mass (low muscle mass or normal), physical perform (“ADL/IADL without loss” or “ADL/IADL loss”), BMI (underweight or normal or “overweight or obesity”), drinking (never smoked or currently smoking or quit drinking), smoking (never smoked or currently smoking or quit drinking), history of hypertension (no or yes), dyslipidemia (no or yes), diabetes (no or yes), and kidney disease (no or yes) except the subgroup variable itself

## Discussion

This is a nationally representative prospective study designed to investigate the relationship between HGS and CVD outcomes in middle-aged and older adults. This study showed that higher levels of handgrip strength are significantly associated with a lower risk of developing cardiovascular disease, including its major components, heart disease and stroke, where there is an inverted U-shaped relationship between HGS and stroke risk. When HGS was higher than 26.13 kg, the multivariable-adjusted HR for stroke risk was reduced by 8.3% per 1 kg increase in HGS. The results were essentially similar across age, sex, BMI, and whether or not they smoked, drank alcohol, were hypertensive, diabetic, hyperlipidemic, and kidney disease populations. It is worth noting that the ‘high’ levels of grip strength we observed were relative to the distribution of our study population and not based on any absolute criteria. This relative categorization approach allows us to explore the relationship between grip strength and CVD risk without relying on external criteria that may vary by population characteristics.

The association between HGS and CVD was made considering different covariates, ranging from basic demographic characteristics to a complex history of chronic disease. The association was slightly weakened but still significant as different variables were added. This stepwise analysis not only confirmed the robustness of HGS as an independent predictor of CVD risk, but also revealed that socioeconomic status, lifestyle, and chronic diseases may play a partial moderating role in this relationship. Nevertheless, even after full adjustment, HGS remained an important indicator of CVD risk, highlighting its potential value in clinical risk assessment.

### Comparison with similar studies

Before our study, Leong et al. [8] found that higher HGS was associated with a lower risk of all-cause mortality and cardiovascular disease in a multicenter study involving 17 countries. This study also showed that grip strength is an independent predictor of CVD, even more so than blood pressure. However, this study focused on a global multinational population, whereas our study focused on middle-aged and elderly people in a Chinese community, providing more targeted regional data. Similarly, Jang SK et al. [21] from South Korea analyzed 8494 middle-aged and older adults aged 45 years or older in the Korean Longitudinal Study of Aging and found that among male participants, the odd ratios corresponding to the risk of CVD in 2nd quartile, 3rd quartile, and 4th quartile compared with the lowest quartile of HGS were 1.365 (95% CI: 1.207, 1.550), 1.141 (95% CI: 1.010, 1.291), and 1.126 (95% CI: 1.001,1.266), respectively, and a consistent negative association was found in women.

In addition, Celis-Morales et al. [22] analyzed the relationship between HGS and CVD based on UK Biobank data. They similarly found that higher HGS was associated with lower CVD risk, but did not explore the inverted U-shaped relationship between HGS and stroke risk in detail. We further refined the relationship between HGS and specific types of CVD (e.g., heart disease and stroke) and equally found an inverted U-shaped relationship between HGS and stroke risk, i.e., both too low and too high HGS may increase the risk of stroke. This finding is inconsistent with the findings of Li et al. [23], who reported a linear relationship between HGS and CVD in all 3 prospective cohorts, with individuals with higher HGS having a lower risk of stroke. In addition, our study noted a specific numerical relationship between HGS and stroke risk of an 8.3% reduction in stroke risk per 1 kg increase in grip strength when HGS exceeded 26.13 kg. This result provides a specific reference standard for clinical practice, similar to the meta-analysis by López-Bueno R et al. [24], whose results also provide specific HGS thresholds for assessing the risk of cardiovascular-related mortality.

Notably, our study covered a wide range of possible confounders, including age, sex, BMI, smoking, alcohol consumption, hypertension, diabetes, hyperlipidemia, and kidney disease. This result suggests that HGS may be a predictor of CVD risk independent of traditional risk factors. In contrast, the study by Silventoinen et al. [25] did not cover such a wide range of population characteristics, although it also considered a variety of confounding factors, which makes our results more applicable to different populations.

### Possible explanations and implications

In studies exploring the relationship between HGS and CVD risk, we have found that high HGS levels are associated with low CVD risk. This finding has multiple possible explanations and has important public health and clinical implications.

HGS, as a simple and easily measured indicator of physical function, may reflect the state of overall muscle mass and skeletal muscle function [26]. A decline in muscle mass and function is commonly associated with the aging process and increased risk of several chronic diseases. Higher HGS may reflect a better state of generalized muscle health, which is associated with a lower risk of CVD [27]. Second, HGS may be associated with physical activity levels. Individuals with high HGS are often accompanied by adequate physical activity, the latter of which has been widely shown to have a protective effect on cardiovascular health by improving lipid profiles, lowering blood pressure, and enhancing insulin sensitivity [28]. In addition, HGS is an important index for measuring nutritional status. A higher HGS may reflect favorable nutritional intake and metabolic status, and poor nutrition is associated with an increased risk of CVD. For example, inadequate protein intake may lead to decreased muscle mass, which increases CVD risk [29].

Although our research found an inverted U-shaped relationship between HGS and stroke risk, the *P*-value overall was lower than 0.05. This finding aligns with some previous studies that have demonstrated the protective effects of muscular strength against CVD [30]. The mechanisms underlying this relationship are likely multifaceted. At the lower end of HGS (< 26.13 kg), increased stroke risk may be attributed to overall frailty, sarcopenia, or poor cardiovascular health [31]. In addition, lower HGS is associated with higher levels of inflammatory markers such as C-reactive protein, and inflammation is a known risk factor for stroke [32]. The protective effect of moderate to high HGS (> 26.13 kg) could be explained by its reflection of overall physical fitness, engagement in regular physical activity, and better cardiovascular health [8]. However, it is important to note that this specific value may vary according to population characteristics (e.g., age, gender, ethnicity) and should not be considered a universally applicable threshold. Future studies should further explore the mechanism of this nonlinear relationship and validate this finding in different populations. In the meantime, we also need to consider confounding factors that may influence this relationship, such as age, gender, and overall health status.

From a public health and clinical perspective, HGS measurement can be widely applied in community and clinical settings to help identify high-risk populations and develop personalized intervention strategies. Regular HGS measurements can early detect individuals with declining muscle mass and function, enabling targeted interventions through nutritional support and exercise training programs to reduce CVD risk. In CVD prevention and management programs, regular HGS measurements can assess the effectiveness of interventions and adjust and optimize them based on measurement results.

In conclusion, our research findings not only reveal the relationship between HGS and CVD risk but also provide a new perspective on the application of HGS measurement in public health and clinical practice. Future studies should delve into the biological mechanisms between HGS and CVD. First, the relationship between HGS and inflammatory markers (e.g., C-reactive protein, interleukin-6), which are closely associated with CVD, should be investigated [33]. Second, examining the association of HGS with oxidative stress and endothelial function may reveal how muscle strength affects vascular health [22]. In addition, exploring relationships between HGS and metabolic regulation (e.g., insulin sensitivity, lipid metabolism) may elucidate the metabolic role of muscle strength in cardiovascular health [34]. Finally, longitudinal study designs and genomic approaches may help to determine the causal and genetic basis of the relationship between HGS and CVD [35]. These lines of research will contribute to a comprehensive understanding of the biological basis of HGS as an indicator of cardiovascular health and provide a scientific basis for prevention and intervention strategies.

### Strength and limitations

This study utilized large-scale, population-based Chinese cohort data to not only focus on the direct association between HGS and CVD, including its major components, heart disease, and stroke, but also considered various potential confounding factors such as age, gender, smoking, alcohol consumption, BMI, and comorbidities, making our analysis results more robust and reliable. The longitudinal follow-up design helped to reveal the causal relationship between the two, rather than just an association. Despite the strengths of this study, there are some limitations. Firstly, while HGS measurement can reflect overall muscle strength, it may not comprehensively assess all risk factors for CVD. The occurrence of CVD involves multiple complex biological mechanisms and environmental factors, and relying solely on HGS measurement may not fully predict an individual’s cardiovascular disease risk. Nevertheless, some studies indicate a negative correlation between HGS and CVD-related indicators. Secondly, the data in this study primarily came from the Chinese population, and although the sample size was large and representative, the results may not be entirely applicable to other ethnicities or regions. Therefore, future research should validate these findings in populations of different races and regions. However, similar results have been reported based on data from multiple populations, including cohorts in South Korea and the UK. Thirdly, while the HGS measurement method used in this study was standardized, it may still be influenced by individual differences such as hand diseases and recent activity levels. These factors could lead to variability in measurement results, thereby affecting its accuracy as a predictor of CVD [36]. Lastly, despite considering various potential confounding factors, there may still be unmeasured or unknown confounding factors such as physical activity, genetic factors, and socioeconomic status, which could impact our research findings [33]. Not only that, but although we indirectly considered possible medication history by adjusting for several major chronic conditions (e.g., hypertension, diabetes mellitus, renal disease, and hyperlipidemia), this approach could not fully capture the complexity and diversity of medication use. This limitation may have affected our interpretation of some associations, especially in cases where medications may directly affect HGS or CVD risk. Future studies should place greater emphasis on the completeness of medication history data and take steps to minimize missing data for more comprehensive and accurate analyses.

## Conclusion

In conclusion, this population-based cohort study demonstrates a close association between HGS and CVD risk, including heart disease and stroke risk, with a possible nonlinear relationship between HGS and stroke. This study suggests that HGS may be a promising measurable biomarker for predicting CVD, providing a more scientifically sound and straightforward basis for CVD prevention.

## Electronic supplementary material

Below is the link to the electronic supplementary material.

Supplementary Material 1

## Data Availability

Publicly available datasets were analyzed in this study. This data can be found here: https://charls.pku.edu.cn/.
